# Global Health Solidarity: A Multidimensional Framework

**DOI:** 10.1111/bioe.70042

**Published:** 2025-10-27

**Authors:** Yijie Wang

**Affiliations:** ^1^ Institute of Technology Ethics for Human Future Fudan University Shanghai Shanghai China; ^2^ Research Centre ‘Normative Orders' Goethe University Frankfurt Frankfurt am Main Hessen Germany

**Keywords:** global health, institutional, moral, prudential, sociopolitical, solidarity

## Abstract

Solidarity has emerged as a vital concept in bioethics. In recent years, the concept of solidarity has transcended domestic boundaries, with its rhetorical power being leveraged across diverse global health contexts. However, despite its prominence in bioethics and its rhetorical use in global health, health solidarity remains largely confined to domestic contexts. This paper fills this gap by exploring the possibility of extending health solidarity on a global level. Rather than pursuing a singular, unitary concept, I propose to conceptualize global health solidarity (GHS) with a multidimensional framework that encompasses four essential modes: prudential, moral, sociopolitical, and institutional. The prudential mode provides a compelling foundation for GHS through self‐interested motivations, emphasizing global health interdependence. The moral mode frames GHS as morally right or good, grounded in relational personhood, functioning either as a duty or a virtue. The sociopolitical mode conceptualizes GHS as a politically significant, prosocial phenomenon in pursuing liberation and confronting injustices, enabling project‐based collaboration beyond identity boundaries. The institutional mode formalizes GHS through established structures, shaping global bioethics frameworks and health governance systems. Recognizing these diverse sources, contexts, and practices of solidarity, the multidimensional framework offers a comprehensive conceptual map for understanding and operationalizing GHS. It provides both analytical clarity and practical guidance for navigating solidarity‐based practices in global health contexts. When compared with established frameworks, such as global health justice, global health governance, global health activism, and global health security, GHS provides distinct added value and deserves a more fundamental place in the current global health discourse.

## 1 Introduction

Solidarity has emerged as a vital concept in bioethics. It is defined as “an enacted commitment to carry ‘costs’ (financial, social, emotional or otherwise) to assist others with whom a person or persons recognise similarity in a relevant respect” [1, p. 52]. At its core, the principle of “standing up beside” [2] emphasizes the interconnectedness of human health, the needs of vulnerable populations, and social responsibility and care. It complements traditional bioethical frameworks focused on individualism and autonomy [3], manifesting in practices like health database governance and organ donation systems [1].

In recent years, the concept of solidarity has transcended domestic boundaries, with its rhetorical power being leveraged across global health contexts from addressing health workforce migration [4], global health research [5, 6], to responses to pandemics [7]. The COVID‐19 pandemic particularly amplified calls for solidarity through the World Health Organization's Solidarity Call to Action for equitable global access to COVID‐19 health technologies [8], the COVID‐19 Solidarity Response Fund [9], and the COVAX facility, which operates under the slogan that “no one is safe, unless everyone is safe” [10].

However, the rhetorical deployment of solidarity in global health contexts often functions more as aspirational language than an actionable framework. For example, while the WHO's Solidarity Call to Action garnered significant attention and symbolic support, it failed to generate binding commitments for technology transfer or patent waiving that would have operationalized its solidarity rhetoric. Similarly, COVAX's solidarity slogan coexisted with continued vaccine nationalism, where wealthy countries secured multiple doses per capita while low‐income countries remained largely unvaccinated.

The gap between rhetoric and implementation reflects both empirical constraints and deeper conceptual limitations in extending health solidarity globally. Empirically, health policy remains primarily within the nation‐state's competence. As Wolf observes, insufficient international institutions and ongoing conflicts create structural barriers to transnational solidarity initiatives [11]. Following this, Prainsack and Buyx concluded that “possibilities of mobilising solidarity in pandemics… are limited” [12], p. 78] with their framework primarily addressing welfare systems within nation‐states rather than transnational applications. Conceptually, many scholars argue that solidarity's core characteristics resist global extension. Rorty, for example, argues that universal solidarity based on common humanity is “weak, unconvincing” [13, p. 191], while Heyd contends that solidarity is “necessarily exclusive, presupposing the existence of competing causes” [14]. This inherent partiality leads Butler and Derpmann to conclude that solidarity cannot ground universal healthcare [15, 16].

This paper aims to address this gap by exploring the possibility of extending health solidarity on a global level. Rather than pursuing a singular, unitary concept, I propose to conceptualize global health solidarity (GHS) with a multidimensional framework that encompasses four essential modes: prudential, moral, sociopolitical, and institutional. Each mode represents a distinct facet of GHS with unique conceptual foundations, normative justifications, practical challenges, and real‐world applications. The multidimensional framework offers a comprehensive conceptual map for understanding and operationalizing GHS. It provides both analytical clarity and practical guidance for navigating solidarity‐based practices in global health contexts. When compared with established frameworks, such as global health justice, global health governance, global health activism, and global health security, GHS provides distinct added value and deserves a more central place in current global health discourse.

This paper unfolds in six sections. Section 2 establishes the rationale for endorsing a multidimensional framework rather than a singular, unitary concept. Section 3 examines the four modes of GHS: prudential, moral, sociopolitical, and institutional. Section 4 highlights the advantages of this framework. Section 5 positions GHS within broader global health discourse, particularly in relation to global health justice. Section 6 briefly concludes the paper and points out its principal implication.

## 2 Toward a Multidimensional Framework of GHS

Before elaborating on the four modes of GHS, I shall establish the rationale for adopting a multidimensional framework rather than a singular, unitary concept. My analysis builds upon three crucial insights from scholars working on the concept of solidarity.

First, solidarity is normatively dependent [16, 17, 18]. It lacks intrinsic moral value, as evidenced by its operation within morally questionable contexts, for example, a Mafia organization. Instead, solidarity's normative force derives from other foundational concepts such as justice and morality. This dependency does not render solidarity redundant, but rather explains why it becomes valuable in social practices when combined with other normative ideas, and accounts for its varied meanings and applications. Because solidarity is normatively dependent on other concepts, it is *necessary* to endorse a multidimensional framework when conceptualizing GHS.

Second, solidarity is a practical idea [1, pp. 44–48]. It exists primarily as an enacted commitment rather than as an abstract value, ideal, or inner sentiment. This practical dimension underscores the importance of context. While solidarity can be analyzed through a unified analytical framework, as an enacted commitment to bearing costs to assist others, different contexts generate different normative reasons for enacting solidary practices. For example, in moral contexts, solidarity manifests through obligations or virtues motivating assistance to others, while in sociopolitical contexts, it emerges through collective action against injustices. It is thus *plausible* to endorse a multidimensional framework when conceptualizing GHS to capture contextual variations.

Third, solidarity is an elusive idea with various uses. Bayertz, for example, identifies four distinct uses of solidarity: morality, society, liberation, and the welfare state [19]. Expanding on this diversity, Prainsack and Buyx identify at least eight intellectual traditions that have shaped our understanding of solidarity: classical sociology, contemporary political thought, communitarianism, care ethics and feminism, rational choice theory, contractualism, global theory, and agonistic theory [12, pp. 97–122]. Further illustrating this conceptual richness, the Global Health Solidarity Project advocates for a “pluriversal” approach that incorporates various cultural and religious perspectives [20]. With these rich intellectual backgrounds, it is *possible* to endorse a multidimensional framework when conceptualizing GHS.

While a single, unitary concept may help reduce the epistemic burden, it would fail to capture the complex, multifaceted nature of solidarity. Such simplification risks either overgeneralizing the concept of solidarity, rendering it too abstract for practical application, or narrowly focusing on a single dimension, thus missing crucial aspects. A unitary approach also cannot explain why solidarity appeals succeed in some contexts yet fail in others. A multidimensional framework addresses these limitations by providing a comprehensive yet structured conceptual model. In the following section, I elaborate on four essential modes of GHS: prudential, moral, sociopolitical, and institutional. While several existing frameworks acknowledge solidarity's multiple dimensions [1, 12], the multidimensional framework of GHS offers distinctive contributions by (1) explicitly elaborating how this multidimensionality enables solidarity to transcend domestic boundaries; (2) systematically analyzing how prudential, moral, sociopolitical, and institutional modes operate and interact in global health contexts; and (3) providing strategic guidance for how these dimensions can be deliberately emphasized or combined for different global health challenges.

## 3 Four Modes of GHS

### 3.1 Prudential Mode

The prudential mode of GHS operates through self‐interested motivations or actions. It is generally assumed that self‐interest conflicts with solidarity, as solidarity sometimes requires sacrificing personal interests for others or the collective. However, enlightened self‐interest can align with solidarity. Consider the case of the organ donation system: while individuals may not donate organs for their immediate gain—indeed, donation typically involves personal cost or inconvenience—they would contribute to organ donation because it can build a safety net that could benefit themselves or loved ones in the future if needed. In this case, enlightened self‐interest transcends narrow egoism by recognizing that individual ultimate long‐term interests are interdependent with the well‐being of others. This demonstrates how solidarity can align with self‐interested motivations or actions.

The prudential mode becomes particularly valuable in global contexts where international cooperation faces significant challenges. Because international relations often operate through realist reasoning that prioritizes national self‐interest, purely moral appeals for solidarity often fail to motivate sufficient action from nation‐states. Besides, the global system lacks the enforcement mechanisms that enable solidarity like within welfare states. Under this background, the prudential mode can be an alternative motivation that drives global solidarity by pragmatic necessity.

In the context of global health, the prudential mode offers a compelling foundation for GHS precisely because health threats demonstrate clear global interdependence. Several key examples illustrate this. First, infectious disease control reveals how pathogens disregard political boundaries, making cooperative approaches essential for effective disease management even from a self‐interested perspective [21, 22]. Besides, climate change and antimicrobial resistance shift vulnerability patterns globally, creating unexpected similarities between distant populations and pragmatic incentives for developing cooperative health infrastructure [23]. Moreover, health worker migration gives prudential reasons for recipient countries to support source countries' healthcare systems [4]. It can also serve as a starting point or “epistemological catalyst” for recognizing the similarities and shared vulnerabilities between wealthy citizens and migrants [24]. Through these mechanisms, prudential motivations can effectively drive global health solidarity even in the absence of strong moral consensus or enforcement mechanisms.

The prudential argument for GHS, however, also has significant pitfalls. First, it confronts the collective action problem. Although agents may share common interests or goals, solidarity can falter when too many group members prioritize individual profit and immediate satisfaction, creating incentives to free‐ride rather than act collectively for common interests. Second, prudential solidarity often manifests as *ad hoc* cooperation aimed at overcoming specific threats rather than promoting comprehensive common goods. This episodic approach fails to build sustainable solidarity infrastructure for ongoing health challenges. In this process, shared interests remain differentiated and asymmetric, undermining the depth and durability of solidarity actions. Third, the prudential mode faces an inherent risk. When no shared interest exists or is perceived, agents lack motivation for solidarity actions. This explains why diseases primarily affecting developing regions, such as malaria, tuberculosis, and neglected tropical diseases, fail to generate comparable international solidarity to pandemics with global reach. These aspects reveal the limitations of the prudential mode of GHS and suggest the need for a complementary mode to achieve comprehensive global health outcomes.

### 3.2 Moral Mode

The moral mode conceptualizes GHS as morally right or good, independent of whether it promotes self‐interest. This normative foundation derives from a relational ontology of personhood—a view that understands individuals not as autonomous, self‐contained entities but as fundamentally constituted through their relationships with others [25]. This relational understanding generates two distinct moral frameworks: a deontological approach, which establishes binding moral obligations for solidarity that may be institutionalized through legal or political mechanisms [26]; and an axiological perspective, which frames solidarity as a supererogatory duty, virtue, or ideal functioning as the “putty” for justice [1]. Additionally, the moral mode engages moral sentiments, such as empathy, trust, and loyalty. While these affective sentiments are neither necessary nor sufficient conditions for solidary action, they strengthen solidarity commitments.

Moral arguments for GHS gain strength as human relationships increasingly globalize. Both deontological and axiological frameworks provide pathways for extending health solidarity globally. From a deontological perspective, our embeddedness in global relationships establishes moral obligations toward distant others. Recent scholarship from African perspectives justifies GHS as a deontological duty derived from the relational concept of personhood embodied in “ubuntu” [27, 28]. This approach becomes particularly compelling when considering shared health vulnerabilities. If moral duties to protect vulnerable populations exist within national welfare states, it is imperative to extend these obligations globally to acknowledge our shared vulnerabilities to health threats [29]. From an axiological perspective, the non‐obligatory nature of solidarity creates space for individuals and communities to voluntarily cultivate solidarity with others across borders, as exemplified by international HIV/AIDS campaigns.

The moral mode of GHS significantly complements alternative approaches in several crucial ways. It provides sustained motivation that persists when prudential calculations might prioritize narrow self‐interest. For marginalized populations or future generations whose reciprocal contributions may be minimal, moral reasoning justifies extending solidarity beyond strategic exchange. Furthermore, it guides institutional design toward equitable outcomes rather than mere efficiency and critically identifies and challenges underlying power imbalances and historical injustices. Owing to these virtues, moral mode offers a robust normative foundation and motivational resources for sustained commitment to GHS.

Nevertheless, the moral mode encounters significant challenges. Deontological approaches may struggle with specifying precise duties owed to distant others, determining responsibility allocation, and establishing effective enforcement mechanisms. Additionally, conflicts between competing obligations inevitably arise. When duties toward compatriots conflict with those toward distant others, deontological frameworks must provide compelling arguments against reasonable partiality. Axiological approaches face different obstacles, particularly regarding motivation. Since axiological solidarity represents an aspirational ideal rather than a strict duty, sustaining a consistent commitment to global health concerns without an immediate personal connection could be challenging in practice.

### 3.3 Sociopolitical Mode

The sociopolitical mode of GHS unfolds across two interconnected dimensions. First, it conceptualizes solidarity as a social phenomenon characterized by prosocial, bottom‐up organizational structures, exemplified in diverse social movements from 18th to 19th‐century labor movements to contemporary feminist and climate change movements [30, 31].^1^ Second, these solidarity practices have a political significance because they often pursue liberation and justice, while fighting against oppression and injustices. Political solidarity, as defined by pioneering scholar Scholz, constitutes “a relation that forms a unity of individuals each responding to a particular situation of injustice, oppression, or social vulnerabilities” [32]. Yet political solidarity extends beyond mere reactivity to encompass affirmative claims within bioethics and public health. It functions to dismantle structural barriers to equitable and universal healthcare [33], advocate for equal access to health resources [34], and prioritize vulnerable populations to achieve health equity [35].

The sociopolitical mode holds significant promise for extending health solidarity globally. It transcends traditional identity‐based boundaries in favor of project‐led collaboration. Unlike solidarity founded on pre‐existing relational ties or national affiliations, sociopolitical solidarity mobilizes around specific goals, enabling cross‐border coalitions addressing shared health challenges. Gould, for example, differentiates between “unitary solidarity”, typically confined to citizen relationships within state boundaries, and “networking solidarity”, which emerges when diverse groups collaborate across borders on projects confronting oppression or exploitation [33, 36]. GHS thus demands “active participation in social movements aimed at improving healthcare nationally or transnationally, or, more defensively, to protesting structural injustices that lead to the wrongful denial of healthcare or to deep inequalities in its allocation” [33, p. 548]. This mode has demonstrably facilitated transnational health advocacy, as evidenced in the HIV/AIDS movement, where organizations like ACT UP united diverse international communities in campaigns for research funding and treatment accessibility [37]. More recently, the People's Vaccine Campaign exemplifies contemporary GHS through its coalition of transnational actors advocating for intellectual property waivers for COVID‐19 vaccines, treatments, and diagnostics, illustrating how project‐focused solidarity can transcend national interests to pursue global health equity [38].

The sociopolitical mode for GHS becomes particularly vital in contexts where institutionalized mechanisms for universal or equitable healthcare are absent or inadequate. These solidarity movements generate substantial mobilizing power that transcends traditional boundaries, facilitating cross‐border collaboration organized around shared objectives rather than pre‐existing affiliations. Such movements strengthen global civil society networks and exert normative pressure on institutional policies and practices within global health governance.

Nevertheless, the sociopolitical mode confronts significant challenges. Practical implementation often encounters resistance from entrenched power structures that prioritize established interests over transformative change. Additionally, bottom‐up movements may struggle with the complex coordination required across diverse healthcare systems and cultural contexts. The episodic nature of solidarity campaigns can limit their capacity to address persistent global health inequities that demand sustained institutional commitment and structural reform.

### 3.4 Institutional Mode

The institutional mode of GHS conceptualizes solidarity as formalized in social institutions. This corresponds to what Prainsack and Buyx identify as the tier 3 level of solidarity, where the values or principles enacted and emerged through interpersonal or group‐based practices are solidified in the contractual, legal, or administrative norms [1]. Modern welfare states, progressive taxation, and health insurance systems are typically considered symbols of institutionalized solidarity, as these systems are designed to share the risks and ensure basic well‐being for all their members.

While European welfare states frequently invoke solidarity as their foundational principle, there exists a problem regarding whether justice, rather than solidarity, provides the more appropriate normative framework for analyzing healthcare and welfare systems. Rawls' influential work established principles for just institutional arrangements [39], which Daniels subsequently extended specifically to healthcare contexts [40]. By contrast, solidarity, or what Rawls termed “fraternity”, has occupied a more peripheral position in democratic theory, which is “unrealistic to expect between members of the wider society” [39, p. 90]. This limitation has prompted scholars like Wolf to question solidarity's role in the distribution of healthcare resources [11]. Nevertheless, Rawls acknowledges that his difference principle inherently embodies fraternal values. Under this principle, individuals accept advantages only when these also improve conditions for the less fortunate, reflecting a form of institutional solidarity [39, p. 90]. I will return to the relationship between justice and solidarity, and particularly GHS and global health justice, later in Section 5.

Despite theoretical debates, the institutional mode of GHS has gained traction in global bioethics frameworks and continues to influence international health governance structures. Even though there is no world state functioning as a counterpart to nation‐states for global redistribution, numerous intergovernmental organizations and legal frameworks effectively express GHS in institutional forms. The World Health Organization serves as a central coordinator of international health initiatives, pandemic responses, and global disease surveillance systems. Similarly, the GAVI Alliance demonstrates institutional solidarity by pooling resources from countries and private donors to systematically improve vaccine access worldwide [41]. Besides, the Universal Declaration on Bioethics and Human Rights (UDBHR) explicitly incorporates solidarity as a core value while embodying solidaristic principles [42]. The WHO Pandemic Agreement represents another emerging institutional mechanism designed to formalize solidarity commitments for pandemic preparedness and response across borders [43].

The institutional mode of GHS, however, may be subject to governance limitations like bureaucratic inefficiencies and frequently become arenas where geopolitical interests compete for influence and control. National self‐interests consistently restrict countries' willingness to enter binding commitments that prioritize collective health outcomes over domestic considerations. Furthermore, power asymmetries between developed and developing nations fundamentally shape how solidarity mechanisms function in practice, typically reflecting and reinforcing existing geopolitical hierarchies rather than transforming them. These structural impediments can undermine the potential of the institutional mode.

## 4 Advantages of the Multidimensional Framework

Through the prudential, moral, sociopolitical, and institutional modes, the multidimensional framework of GHS offers several significant benefits for understanding, fostering, and implementing solidarity in global health contexts.

First, the multidimensional framework of GHS provides analytical clarity. It recognizes that solidarity does not emerge from a single source or motivation. Instead, the framework bridges different theoretical traditions in bioethics, political philosophy, and international relations that have historically approached solidarity from different angles. It also recognizes that solidarity is normatively dependent and can manifest in various forms depending on context. By distinguishing between the prudential, moral, sociopolitical, and institutional dimensions, we gain a clearer understanding of the complex dynamics that drive or inhibit global health cooperation.

Second, the multidimensional framework of GHS offers strategic appeal to diverse stakeholders. Different stakeholders may respond to different motivations. Some actors may be more responsive to prudential arguments, while others are motivated by moral considerations, sociopolitical projects, or institutional commitments. The multidimensional framework enables tailored communication strategies that can appeal to diverse stakeholders. When addressing political leaders, it might be more effective to emphasize prudential aspects. When engaging in civil society, the sociopolitical mode may resonate more strongly. When working with global institutional partners, institutional dimensions could be highlighted.

Third, the multidimensional framework helps explain why solidarity appears strong in some contexts but weak in others. When solidarity efforts fail, the multidimensional framework provides a more nuanced diagnostic tool. Rather than simply concluding that “solidarity failed”, we can identify specific dimensional weaknesses by asking questions like: Was self‐interest insufficiently aligned with collective action? Were moral obligations not clearly articulated or accepted? Did social connections prove too weak? Were institutional mechanisms inadequate? This diagnosis enables more targeted interventions.

Finally, the multidimensional framework of GHS provides the foundation for illuminating the various relationships between different modes. The four modes can be complementary to each other, each capturing a different facet of how solidarity functions in global health contexts. They can be compensatory when another is weak: where moral motivation is insufficient, institutional mechanisms can create accountability. They can reinforce one another, as sociopolitical movements may strengthen moral commitments. They can even dynamically evolve to another, as initial cooperation based on prudential interests might gradually develop social bonds. Or they can be in conflict, as prudential calculations sometimes conflict with moral obligations. While partial or dimensional forms of solidarity have been studied, further research on their relationship is needed to develop a more comprehensive understanding and implementation mechanism toward full GHS.^2^

## 5 GHS in Global Health Discourse

In the current global health discourse, scholars and practitioners are more familiar with frameworks like global health justice, global health governance, global health activism, and global health security. What added value, then, does the concept of GHS provide? How can it advance our current global health discourse? In the following, I will discuss the positive content and the added value of GHS in comparison with these established frameworks.

Compared with *global health justice*, which focuses on the fair distribution of health resources and is concerned with structural factors causing health disparities, GHS offers distinct added value.

First, GHS motivates global health justice. While Rawls established justice, rather than solidarity, as a fundamental value to social institutions, this primacy encounters practical limitations. Communitarian philosopher Sandel persuasively argued that providing certain goods requires a sense of solidarity, without which citizens would not make the sacrifices demanded by the difference principle [44]. Besides, many cosmopolitan theorists view solidarity as a motivational force to fulfill cosmopolitan duties of justice [29]. The multidimensional framework of GHS developed here extends this understanding by showing how solidarity provides prudential, moral, and sociopolitical motivation for global health justice. These motivational dimensions do not operate for solidarity's sake, as solidarity itself remains normatively dependent on ideas like justice, but can serve to enable and sustain the practical pursuit of just distribution of healthcare goods globally.

Second, GHS mobilizes global health justice. Many sociopolitical philosophers position solidarity as the social basis for justice. Krishnamurthy, for example, argues that solidarity supports justice in a developmental way because commitment to justice is learned through relational engagement with others [34]. Similarly, Gould maintains solidarity as a prerequisite for justice, emphasizing that solidary practices create concrete spaces for the mobilization of justice [45, pp. 119–131]. Through solidary practices, the sense of justice is cultivated, practiced, and mobilized across multiple domains, addressing not only distributive justice but expanding to encompass productive, reproductive, and structural dimensions of global health justice.

Third, GHS constitutes global health justice. Building on the previous point, Gould further argues that solidarity constitutes justice by establishing unity within broad transnational associations where redistribution becomes justified [46]. When people develop solidarity across borders, they create a shared context that justifies redistributive actions. This relationship is evident in Rawls' difference principle, which itself expresses the idea of fraternity as a justification for addressing inequalities. Munoz‐Dardé further expands this through “contractualist fraternity,” proposing that in a just system, every constraint must be justified to each individual member of a solidarity community [47]. This reflexive character of solidarity, where mutual recognition and reciprocal justification are centered, constitutes justice by creating the social conditions where principles of justice can be meaningfully articulated, accepted, and implemented across diverse global contexts.

Fourth, GHS complements global health justice. While justice concerns rights and duties derived from justice principles, solidarity emphasizes the relational aspects of cooperation and commonality [46, 48, pp. 71–108]. In Habermas' terms, justice and solidarity are “two sides of a coin” [49]. While justice concerns equal freedoms of individuals, solidarity addresses the welfare of citizens who are intimately linked in an intersubjectively shared form of life. This complementary relationship helps address global health challenges with a more comprehensive perspective.^3^

Compared with *global health governance*, which focuses on the institutional arrangements for managing health issues and addresses policy‐making processes and implementation, GHS complements global health governance in several meaningful ways. It provides the moral foundation for global health governance, originating from human relationships and the interconnectedness of health rather than institutional arrangements. It fills the institutional gaps: when formal governance mechanisms are weak, slow, or absent, solidarity drives informal cooperation that can respond to immediate needs. These solidary movements often have public support and can put normative pressure on existing governance structures toward transformation.

Compared with *global health activism*, which focuses on advocacy and mobilization for health changes from a bottom‐up approach through social movements, GHS provides a complementary framework for addressing global health challenges. GHS emphasizes human relationships and shared vulnerability as common ground for health movements, which can reduce the polarization that activist framing might inadvertently create. It builds on broader coalitions, which can unite diverse stakeholders who might not engage in activist approaches. It also provides the moral foundation for sustained activism beyond immediate campaigns.

Compared with *global health security*, which focuses on protection against cross‐border health threats often driven by self‐interest, GHS provides a more comprehensive framework. GHS builds on broader moral foundations. It extends beyond concerns about self‐interest and national security to genuine care for others. It recognizes shared vulnerability rooted in human relationships and considers all populations worthy of protection, not just those presenting security risks. GHS also addresses diverse health threats, extending concern beyond immediate threats to include non‐communicable diseases, chronic conditions, and everyday health challenges that affect human well‐being globally.

## 6 Conclusion

This paper set out to explore the possibility of extending health solidarity on a global level. It recognizes that solidarity is a normatively dependent idea that builds on different motivations and justifications and manifests in various forms depending on the context. This dependency necessitates the endorsement of a multidimensional framework of GHS rather than a single, unitary concept.

The multidimensional framework developed in this paper encompasses four essential modes: prudential, moral, sociopolitical, and institutional. The prudential mode operates through self‐interested motivations or actions. It offers a compelling foundation for GHS precisely because health threats demonstrate clear global interdependence. The moral mode conceptualizes GHS as morally right or good, deriving from a relational ontology of personhood. It can be either deontological, as a moral duty, or axiological, as supererogatory, offering a robust normative foundation and motivational resources for sustained commitment. The sociopolitical mode conceptualizes solidarity both as a social phenomenon characterized by prosocial, bottom‐up organizational structures and as politically significant in pursuing liberation and confronting injustices. It holds promise for extending health solidarity globally by transcending traditional identity‐based boundaries in favor of project‐led collaboration. The institutional mode conceptualizes solidarity as formalized in social institutions. It has gained traction in global bioethics frameworks and continues to influence international health governance structures. Integrating these dimensions, the multidimensional framework of GHS provides both analytical clarity and strategic appeal to diverse stakeholders, offering a comprehensive framework for analyzing and operationalizing health solidarity across borders.

The principal implication of this paper is that GHS deserves a more central position in current global health discourse. GHS demonstrably provides distinct added value when compared with established frameworks such as global health justice, global health governance, global health activism, and global health security. It motivates, mobilizes, constitutes, and complements global health justice with relational connections that justify and enhance arguments for the fair distribution of healthcare goods globally. It expands the reach and deepens the foundations for global health governance, global health activism, and global health security. As a concept that connects with each of these established frameworks, GHS can serve as an integrative force that helps align these approaches toward more coherent global health responses. The foundational importance of GHS stems from our nature as social beings in relationship with one another. This relational reality creates both the possibility for and necessity of solidarity as a core principle in addressing the inherently interconnected challenges of global health. Recognizing this fundamental aspect of human experience, GHS should be placed in a more fundamental place in global health discourse and practices.

## Conflicts of Interest

The author declares no conflicts of interest.

## Endnotes

1 It is worth noting Durkheim's distinction between mechanical and organic solidarity. While mechanical solidarity is based on similarity, shared beliefs, and collective consciousness in traditional societies, modern societies are characterized by organic solidarity based on interdependence and complementary differences made by the division of labor [50]. This distinction demonstrates how solidarity can emerge through different mechanisms that correspond to different aspects of the sociopolitical mode. While mechanical solidarity's emphasis on shared identity and values complements the discussion of solidarity based on common vulnerability, organic solidarity's focus on interdependence supports the arguments about mutual dependence in global health systems.

2 Scholz's recent concept of “scaffolded solidarity” offers valuable insights. Rather than operating as a fixed, bounded concept, solidarity functions like a scaffold composed of various parts that can be shaped and reshaped depending on situational needs [51]. This scaffolded concept validates the argument that solidarity need not be confined to a single definitional framework or bounded by traditional membership criteria. Instead, just as scaffolded solidarity can emphasize different components, the multidimensional model allows for varying emphasis on prudential self‐interest, moral obligations, or sociopolitical commitments depending on what a particular global health challenge requires.

3 These relationships between GHS and global health justice do not conflict with the claim that solidarity is normatively dependent. Solidarity can serve these functions precisely because we are social beings in relationship with one another. This allows it to both derive normative weight from and contribute to broader moral frameworks.
